# Acu‐URO17 is a highly sensitive and specific bladder cancer biomarker

**DOI:** 10.1002/bco2.338

**Published:** 2024-02-29

**Authors:** Liwu Guo, Alexandra Cid, John Cucci, Brian Kunkel, Lisa Defeis, Michael Matthews

**Affiliations:** ^1^ Acupath Laboratories Inc Plainview New York USA

**Keywords:** Acu‐URO17, bladder cancer, cytology, fluorescence in situ hybridization, negative predictive value, urothelial carcinoma, voided urine

## Abstract

**Objective:**

This study evaluates the efficacy of Acu‐URO17, a highly sensitive and specific immunocytochemistry (ICC) test targeting Keratin 17, in comparison to urine cytology and UroVysion™ fluorescence in situ hybridization (FISH) for detecting bladder cancer cells in voided urine specimens.

**Methods:**

Acupath conducted a large‐scale comparison study using 2378 voided urine specimens. Acu‐URO17, urine cytology and UroVysion™ FISH were performed on these specimens according to standardized protocols. Sensitivity, specificity, positive predictive value (PPV) and negative predictive value (NPV) were calculated for Acu‐URO17 in comparison to urine cytology and UroVysion™ FISH.

**Results:**

In cases diagnosed with high‐grade urothelial cancer via urine cytology, Acu‐URO17 demonstrated a sensitivity of 96% and a specificity of 82%. When compared to UroVysion™ FISH results, Acu‐URO17 exhibited a sensitivity of 97.1% and a specificity of 77.8%, surpassing the sensitivity of UroVysion™ FISH (57.1%). Notably, Acu‐URO17 showed a high NPV of 99.9%, indicating its reliability in confirming negative urine cytology results and risk‐stratifying atypical and suspicious cytology results.

**Conclusion:**

The results of this large‐scale prospective study support Acu‐URO17 as a clinically relevant, non‐invasive and cost‐effective tool for detecting bladder cancer cells in voided urine specimens. Its high sensitivity, specificity and NPV make it a valuable adjunct to urine cytology and UroVysion™ FISH in the diagnosis and management of urothelial carcinoma (UC).

## 1 INTRODUCTION

According to the American Cancer Society recent data as of 2023, bladder cancer is the fourth most frequent cancer in men (approximately 62.420 new cases) and is less frequent in women (approximately 19.870 new cases) annually.^1^ Urothelial cancers account for ~95% of diagnosed bladder cancers,^2^ and most cases are diagnosed as Non‐Muscle Invasive Bladder Cancer (NMIBC),^3^ which provides a treatable prognosis.^4^ NMIBC cases have a high recurrence rate of 80% in high‐risk lesions and up to 50% in low‐risk lesions; the 5‐year survival rate is 94% if detected early.^5^ For patients with urothelial carcinoma (UC), clinical guidelines recommend cystoscopies performed in 3‐month intervals during the first 2 years, 6‐month intervals the following 2 years and then once every year.^5^ Therefore, lifelong surveillance is an important part of routine management for patients with bladder cancer.^6^ Furthermore, since one of the first symptoms of bladder cancer includes hematuria,^7^ invasive cystoscopy is performed on a substantial portion of hematuria patients even though most of these patients might not have UC.^8^

Cystoscopy, in combination with voided urine cytology and upper urinary tract imaging, plays a key role in the surveillance of the recurrence of bladder cancer and detection of new UC in hematuria patients.^5^ However, the utilization of imaging and cystoscopy is often not effective in detecting smaller lesions, and frequent cystoscopy is associated with complications such as a urinary tract infection, hematuria and morbidity.^9^ In addition, many of the patients required to undergo these painful and expensive procedures even though they may not have active UC.^5^ Thus, a non‐invasive test that could determine who requires, and as importantly, who does not require cystoscopic follow‐up is a significant unmet clinical need in the management of UC.

Urine cytology is widely used as a non‐invasive method for screening and surveillance of bladder cancers; it is highly specific (~90%) for the detection of UC, but has low sensitivity (~48%) for the detection of UC, especially for low‐grade UC where it misses over half of the UC.^3^ Furthermore, reactive cellular changes associated with infection or inflammation can also induce cellular atypia, mimicking high‐grade UC that could contribute to the general inaccuracy of urine cytology. The recent introduction of the Paris system for urine cytology tried to facilitate the interpretation of the cytology results, but the clinical implications of ‘suspicious’ and ‘atypical’ categories are still not clearly defined which causes significant confusion for physicians.^3^

Currently, there are several urinary biomarker tests commercially available, including Food and Drug Administration (FDA) approved UroVysion™ (Abbott Molecular, Des Plaines IL) FISH test. Other biomarkers include nuclear matrix protein NMP‐22 (Maritech, Newton MA), BTA stat (Polymedco, NY) and BTA TRAK (Bard Diagnstics Redmont, USA).^7^ However, most of these available urinary markers lack sensitivity in detecting early‐stage UC, and the clinical evidence still does not appear to support the widespread application of the tests in clinical settings.

Exploration of Keratin 17 as a biomarker associated with bladder cancer has been recently investigated.^7^ Keratin 17 is normally expressed in stem cells of embryonic ectoderm, skin appendages and the endocervical mucosa, but not in most normal mature epithelia, and is thought to be involved in tissue regeneration and repair.^10^ In 2015, Escobar‐Hoyos et al.^10^ discovered that Keratin 17 functions as an oncoprotein by regulating the subcellular localization and degradation of p27^KIP1^, influencing cervical cancer pathogenesis, which suggested that keratins overexpressed selectively in human carcinomas may offer diagnostic and prognostic utility. Babu et al.^11^ carried out a clinical study to verify Keratin 17's potential as the specific biomarker for bladder cancer. Later studies confirmed that Keratin 17 showed extremely high sensitivity (80–100%) and high specificity (86–96%) on selected urine specimens from urothelial carcinoma from both recurrent UC and new UC from haematuria patients.^11^, ^12^, ^13^

## 2 MATERIALS AND METHODS

Acupath Laboratories obtained the key reagents for Acu‐URO17 biomarker from KDx Diagnostics (Los Gatos, CA) and internally validated it as a laboratory‐developed test. For cytology, Urovysion^TM^ and Acu‐URO17 analysis, 2378 urine specimens were collected between January 2022 and December 2022.

### 2.1 Urine cytology method

Specimens arrived preserved in a ratio of 2:1, Voided Urine to PreservCyt (Hologic) from the clinics to AcuPath. Fifty‐millilitre voided urine samples were centrifuged at 2500 rpm for 5 min, and then, the gently suspended pellets were filtered through polycarbonate membrane filters with 5 μm pores (Costar® filter system, ThinPrep™ 5000 processor, HOLOGIC). Cell monolayers were obtained by gently imprinting the filters onto glass slides. The samples were fixed by immediate immersion in Delaunay fixative (96% ethanol 1:1 + 0.5 ml/L trichloroacetic acid) and then stained with Papanicolaou.

Based on the Paris system diagnostic criteria,^3^ the samples were diagnosed as high‐grade urothelial carcinoma (HGUC), suspicious of high‐grade urothelial carcinoma (SHGUC), atypical urothelial cells (AUC) and negative for high grade urothelial carcinoma (NHGUC). HGUC had a N/C (nucleus/cytoplasm) ratio of ≥0.7; nucleus had moderate to severe hyperchromasia; nuclear membrane was markedly irregular; and chromatin was coarse and/or clumped. SHGUC had a N/C ratio of 0.5–0.7; nucleus had moderate to severe hyperchromasia plus either markedly irregular nuclear membranes or irregular clumpy chromatin. AUC had non‐superficial and non‐degenerated urothelial cells with an increased N/C ratio (>0.5); and changes in nuclear hyperchromasia, irregular nuclear membranes or irregular, coarse, and clumped chromatin. NHGUC had benign urothelial, squamous and glandular cells; benign urothelial tissue fragments; and changes associated with stones, or viral cytopathic effects, due to polyoma virus or post‐therapy effects.

### 2.2 Acu‐URO17 immunocytochemistry method

Samples were centrifuged at 1000×*g* for 10 min; each pellet was resuspended in 20 ml of PreservCyt (Hologic) and then transferred to charged‐glass slides using a T‐5000 (Hologic) cell processor. The slides were stained using a Link 48 Autostainer (Agilent Technologies). Endogenous peroxidase activity was blocked using the EnVision FLEX wash peroxidase‐blocking reagent (Agilent Technologies). Slides were incubated with anti‐Acu‐URO17 antibody (KDx1 mAb; 1:32 dilution), processed with the direct polymer‐based immunoperoxidase method using EnVision FLEX HRP, developed in EnVision FLEX DAB+ chromogen and counterstained with haematoxylin. Slides were dehydrated with graded ethanol and protected with a cover slip. An Acupath pathologist screened the slides, quantitating the total number of urothelial cells expressing Acu‐URO17 per slide. Acu‐URO17 slides were independently scored, and the number of cells expressing Acu‐URO17 was reported using the following criteria: 0–4 urothelial cells expressing Acu‐URO17 were reported as negative, 5–19 urothelial cells expressing Acu‐URO17 were reported as low expression and over 20 urothelial cells expressing Acu‐URO17 were reported as high expression.

### 2.3 FISH method

FISH for UroVysion^TM^ was performed as described in previous literature.^14^, ^15^, ^16^ In brief, voided urine specimens were collected and handled according to the guidelines established in the package insert provided with the UroVysion™ Bladder Cancer Kit.^17^ Approximately 30–50 ml of voided urine was collected in a 120 ml bottle containing 33 ml of pre‐aliquoted PreservCyt ™ Solution (Cytyc, Londonderry, NH). The specimens were then mixed in a 2:1 ratio with the PreservCyt™ and kept at 4–8°C until ready to be processed.^14^ Samples were then centrifuged at 600×*g* for 10 min, supernatant discarded and the remaining cell pellets mixed with 10 ml of Carnoys fixative (methanol: acetic acid in a 3:1 ratio). The specimens were again centrifuged at 600×*g* for an additional 10 min.

Cells recovered from the voided urine specimens were individually added to a 12 mm circle imprinted on an Ikonisys slide (Ikonisys, Inc, New Haven, CT), using a 10 μl pipette.^14^

Slides were processed using the VP‐2000 Processor (Abbott Molecular, Inc., Des Plaines, IL) and followed the guidelines established by the UroVysion™ Bladder Cancer Kit.^17^ The probe was then added to the slides and sealed using 12 mm coverslips and rubber cement. Hybridization was performed on the Thermobrite™ (Abbott Molecular, Inc., Des Plaines, IL), whereby the slides were warmed to 72°C and then cooled to 42°C. The following day (16–24 h later), slides were washed with a series of detergents and 10 ul of DAPI II applied. Slides were then scanned with the Ikoniscope (Ikonisys, Inc, New Haven, CT), to identify any genetic abnormal cells.^14^

The criteria for positive FISH abnormalities were established as follows: if the scan yielded ≥4 cells with an ‘aneuploid’ signal pattern, ≥12 cells with ‘0 gold’, or ≥10 cells with a ‘single gain’ of one chromosomal locus,^15^ or ≥10 cells with a ‘tetraploid/near‐tetraploid’.^16^

## 3 RESULTS

The study used the cytology test results as the reference to evaluate the sensitivity and specificity of the Acu‐URO17 test results on UC and NHGUC (Table 1). Acu‐URO17's sensitivity was 95.92%, and specificity was 82.35%. The positive predictive value (PPV) was 10.26% (47/[47 + 411]), and the NPV was 99.9% (1918/ [1918 + 2]). The cytology test results were also used as the reference to evaluate the detection rates of Acu‐URO17 on SHGUC and AUC samples. Seventy four out of 82 cytology samples confirmed SHGUC cases were detected as Acu‐URO17 positive (90.24%); among them, 76.83% were Acu‐URO17 high‐expression, 13.41% were Acu‐URO17 low‐expression; 323 out of 469 cytology samples confirmed AUC cases were detected as Acu‐URO17 positive (68.87%); among them, 46.06% were Acu‐URO17 high‐expression, and 22.81% were Acu‐URO17 low‐expression.

**TABLE 1:** Detection rates of Acu‐URO17 on urothelial carcinoma (UC), negative for high‐grade urothelial carcinoma (NHGUC), suspicious for high‐grade urothelial carcinoma (SHGUC) and atypical urothelial carcinoma (AUC) specimen.

Cytology	*N*	Acu‐URO17 Expression (Total)	Acu‐URO17 High Expression	Acu‐URO17 Low Expression	Acu‐URO17 Negative (Total)
UC	49	47 (95.92%)	44 (89.79%)	3 (6.12%)	2 (4.08%)
NHGUC	2329	411 (17.65%)	110 (4.72%)	301 (12.92%)	1918 (82.35%)
SUSPICIOUS	82	74 (90.24%)	63 (76.83%)	11 (13.41%)	8 (9.76%)
ATYPICAL	46	323 (68.87%)	216 (46.06%)	107 (22.81%)	146 (31.13%)

Three methods were used to detect urothelial cancer cells for 1378 out of the total 2378 urine samples presented in Table 2: cytology, Acu‐URO17 and UroVysion™ FISH. Thirty‐four Acu‐URO17 positive results out of 35 confirmed UC cases were detected, showing a sensitivity rate of 97.14%. Out of 1343, 1045 Acu‐URO17 negative results confirmed NHGUC cases were detected, showing a specificity rate of 77.81%. Both sensitivity and specificity of Acu‐URO17 test were highly consistent with their counterparts in Table 1. Twenty‐one FISH positive results out of the 35 confirmed UC cases show sensitivity as 60%, and 1328 FISH negative results out of the 1343 confirmed NHGUC cases show the specificity as 98.89%.

**TABLE 2:** Acu‐Uro17 and UroVysion™ detections on UC.

Cytology	*N*	Acu‐URO17 Positive	Acu‐URO17 Negative	FISH Positive	FISH Negative
UC	35	34 (97.14%)	1 (2.86%)	21 (60%)	14 (40%)
NHGUC	1343	298 (22.19%)	1045 (77.81%)	15 (1.12%)	1328 (98.89%)

In Table 3, 55 Acu‐URO17 positive results out of 61 confirmed SHGUC cases were detected in 55/61 (90.2%) cases; among them, 78.7% were Acu‐URO17 high‐expression, and 11.5% were Acu‐URO17 low‐expression (data not shown). Acu‐URO17 positive results out of 230/330 (69.7%) confirmed AUC cases; among them, 48.2% were Acu‐URO17 high‐expression, and 21.5% were Acu‐URO17 low‐expression (data not shown). In contrast, UroVysion™ showed positive results of 23/61 (37.7%) in confirmed UC cases and 38/330 (11.5%) positive results in confirmed AUC cases.

**TABLE 3:** Acu‐URO17 and UroVysion™ detections on suspicious for high‐grade urothelial carcinoma (SHGUC) and atypical urothelial carcinoma (AUC).

Cytology	*N*	Acu‐URO17 Positive	Acu‐URO17 Negative	FISH Positive	FISH Negative
SUSPICIOUS	61	55 (90.2%)	6 (9.84%)	23 (37.7%)	38 (62.3%)
ATYPICAL	330	230 (69.7%)	100 (30.30%)	38 (11.5%)	292 (88.5%)

## 4 DISCUSSION

In this study, 2378 Acu‐URO17 tests were conducted, about 15–30 times the size of previous clinical trials, representing the largest trial up to date. The limitation of this study was that biopsy and cystoscopy data were unable to be used, due to the biopsy and cystoscopy data being unavailable at the time of the study. Instead, the cytology test was used to provide reference diagnosis and study the correlation between it and the Acu‐URO17 test since the urine cytology test with its high specificity means that all samples that were determined to have positive urine cytology results for UC were almost certain to have active UC. Thus, the positive urine cytology results were used as a gold standard in which to identify samples that had active UC. Based on these criteria, the Acu‐URO17 test showed sensitivity as high as 95.92% (Table 1), consistent with the previously reported results,^11^, ^12^, ^13^ strongly suggesting that Acu‐URO17 test is not only a sensitive method but also a reproducible method in detecting UC in a real clinical setting. In addition, the sensitivities of the Acu‐URO17 test on SHGUC specimens and AUC specimens were also examined. The results showed that Acu‐URO17 test had a sensitivity of 90.24% on SHGUC specimens and a sensitivity of 68.87% on AUC specimens (Table 1), which shows that Acu‐URO17 test can risk‐stratify patients who are high or low‐risk for UC in SHGUC or AUC urine cytology samples. Similarly, Acu‐URO17 test specificity was 82.35% for high‐grade lesions and Carcinoma in situ (CIS) (Table 1), within the range of 86–96% reported before.^11^, ^12^, ^13^

Urine cytology has generally poor performance in detecting UC, especially low‐grade UC.^3^, ^13^ This may generate false‐negative results, therefore lowering the specificity and PPV of the Acu‐URO17 test. In the early stage of carcinogenesis, cytology tests will likely not be able to identify these cells since they may have not yet morphologically changed significantly. In comparison, these initiated or promoted cells still express Keratin 17; therefore, Acu‐URO17 test can detect them reliably even in morphologically normal cells. Babu et al.^12^ reported that the Keratin 17 was more sensitive than cytology for low‐grade and high‐grade for UC. However, a limitation of the study was when cytology results are treated as the standard for true negative (NHGUC), the higher detection rate by Acu‐URO17 test would generate more false‐positive results and drive down the specificity of Acu‐URO17 test. This may explain why the specificity for the Acu‐URO17 study was relatively low (82.35%) (Table 1) compared to the previously published studies.

In addition to the sensitivity and specificity, the NPV (99.90%) and PPV (10.26%) were calculated (Table 1). The 99.90% NPV strongly demonstrates that the Acu‐URO17 test can reliably detect truly ‘benign’ specimens as negative since Keratin 17 is not expressed. The 99.90% NPV carries an important value to accurately assure that negative results can help rule out the UC possibility. The low PPV value is probably due to the high‐false positive results resulting from using cytology negative NHGUC samples as the true negatives in the analysis.

In Table 2, 1343 out of 1378 samples were detected as negative by cytology exams. However, among these 1343 samples, 298 were detected as Acu‐URO17 positive, and 15 were detected as UroVysion™ FISH positive. It could be especially important to closely follow up on these patients, providing them with further or other diagnostic means. In fact, cytology exam, Acu‐URO17 ICC test and UroVysion™ FISH can all be auxiliary methods to detect specimens that might have been missed by other means, together providing a more accurate diagnosis.

The aneuploidy of chromosomes has been broadly discovered as a distinct characteristic of cancer cells and has been employed as diagnostic means to detect original or recurrent cancer cells. The UroVysion™ FISH test has been successfully detecting bladder cancer cells by identifying the aneuploidy of chromosomes 3, 7 and 17 as well as the loss of both chromosomal 9p21 segments. Among the 2378 Acu‐URO17 tests, 1378 specimens were also tested with the UroVysion™ FISH test. Acu‐URO17 test showed a sensitivity of 97.14% and a specificity of 77.81%, while the UroVysion™ FISH test showed a sensitivity of 57.14% and specificity of 77.22%, indicating that Acu‐URO17 test is extremely sensitive when compared to the UroVysion™ FISH test (Table 2). For the SHGUC cases and AUC cases, Acu‐URO17 test showed sensitivity as 90.16% and specificity as 69.70%, while UroVysion™ FISH showed sensitivity as 36.07% and specificity as 9.39%. While UroVysion™ is a well‐established and widely accepted method, this study explicitly suggests that the Acu‐URO17 test is a reliable method as well.

In summary, this study suggests that the extremely sensitive and specific Acu‐URO17 test can function as a reliable auxiliary test to surveil the recurrence and even provide initial detection of bladder cancer cells in voided urine.

## AUTHOR CONTRIBUTIONS

Liwu Guo and Alexandra Cid were primarily responsible for the background, data analysis and writing of the paper. Lisa DeFeis was primarily responsible for gathering the data sets out of the Acupath LIS and formatting the data to be reviewed. John Cucci and Brian Kunkel were primarily responsible for analysing the data sets and formulating conclusions from the data. Michael Matthews was primarily responsible for correlating the cytology, Acu‐URO 17 and FISH data. Michael has worked with the Acu‐URO 17 Immunocytochemistry test for the past 4 years and worked with the FISH assay for the past 20 years. All of the authors have brought great knowledge and insight to this project.

## CONFLICT OF INTEREST STATEMENT

We the authors declare that we have no financial interest or personal relationships that could have influenced the work reported in this paper.
